# The mosquito-stage transcriptome of the neglected malaria parasite *Plasmodium malariae* reveals species-specific gene expression signatures and functional adaptations

**DOI:** 10.1371/journal.pntd.0014845

**Published:** 2026-11-02

**Authors:** José Luis Ruiz, Bárbara Díaz-Terenti, Thierry Lefèvre, Serge Rakiswendé Yerbanga, Thomas Dan Otto, Elena Gómez-Díaz

**Affiliations:** 1 Instituto de Parasitología y Biomedicina López-Neyra, Consejo Superior de Investigaciones Científicas, Granada, Spain; 2 Functional Genomics Center Zurich, ETH Zurich/University of Zurich, Zurich, Switzerland; 3 Swiss Institute of Bioinformatics, Amphipôle, Quartier UNIL-Sorge, Lausanne, Switzerland; 4 MIVEGEC, IRD, CNRS, University of Montpellier, Montpellier, France; 5 Institut de Recherche en Sciences de la Santé (IRSS), Bobo Dioulasso, Burkina Faso; 6 Bernhard Nocht Institute for Tropical Medicine, Hamburg, Germany; 7 Universität Hamburg (UHH), Hamburg, Germany; 8 School of Infection and Immunity, University of Glasgow, Glasgow, United Kingdom; AstraZeneca plc, UNITED STATES OF AMERICA

## Abstract

**Background:**

To survive and multiply in different host niches, malaria parasites require sets of proteins that are expressed during the life cycle in a timely manner. *Plasmodium malariae* is a human malaria parasite that is present in most malaria-endemic regions. This species remains enigmatic due to the inability to maintain it in long-term culture, the low parasitemia observed in human infections, and its frequent co-infection with other malaria species.

**Methodology/principal findings:**

We investigate the transcriptome of *P. malariae* during transmission in the vector by analyzing RNA-seq data from midgut and salivary gland parasite stages obtained in two experimental mosquito infections with field *P. malariae* isolates. Our analysis identified 3,699 expressed genes, of which 263 are developmentally regulated and 309 are *P. malariae*-specific, including genes with unknown functions and without orthologs in other *Plasmodium spp*. We detected unique expression patterns of the ApiAP2 family of transcription factors, many of which appear to function as master regulators during sporogony. Several members of multigene families, such as *pir*, *phist*, and *fam-l*, were found to be expressed, and new families potentially showing transcriptional heterogeneity were described.

**Conclusions/significance:**

Our findings indicate that the *P. malariae* transcriptome is largely conserved; however, a distinct set of expressed genes lacking detectable orthologs may contribute to differences in transmission and virulence. Given the scarcity of genomic and transcriptomic data for this pathogen and considering the global distribution of *P. malariae* and its potential role in sustaining malaria transmission, this study has direct implications for malaria control and elimination efforts.

## Author summary

Malaria is a globally significant disease caused by parasites of the genus Plasmodium. Of the six species known to infect humans, five are responsible for the vast majority of malaria cases worldwide. In this study, we investigate *Plasmodium malariae*, one of the least studied human malaria parasites. Research on this species has been limited because infections often go undetected owing to low parasite densities, and the inability to maintain the parasite in long-term culture has impeded detailed investigations of its biology and transmission. We investigated its transcriptome as the parasite develops inside the mosquito, a key stage for its transmission from one person to another. By analyzing gene activity at different time points during development, we identified which genes are turned on, many of which are unique to this species and still have no known function. We also identified genes that may act as key regulators of the process. Taken together, our results indicate that *P. malariae* has unique characteristics that distinguish it from other malaria parasites. Understanding these differences may help explain why it typically causes milder symptoms and provide useful information for improving disease control strategies.

## Introduction

Human malaria is a devastating disease and the cause of a huge global health burden, with 282 million cases of disease and 610,000 related deaths in 2024 [1]. It is caused primarily by six species of protozoan parasites of the genus *Plasmodium*. *P. falciparum* is the most virulent and is the predominant species in sub-Saharan Africa, whereas *P. vivax* is more prevalent in Asia and Latin America. Together, these two species account for the majority of global malaria morbidity and mortality [1–3]. Other human-infecting species remain relatively understudied, such as *P. malariae*, which occurs across most malaria‑endemic regions. This species is frequently undetected due to its low parasitemia and the limited species-specific sensitivity of routine diagnostic tools [4–6].

Recent large-scale genomic surveys have revealed moderate to high prevalence of *P. malariae* infections, with estimates that vary widely between regions, for instance, 2–5% in West and Central Africa [7,8], up to 10% in Southeast Asia [9], and localized outbreaks exceeding 20% [10]. Importantly, these results suggest that the burden of *P. malariae* may be greater than previously acknowledged; however, persistent underdiagnosis, co-infection, and the lack of comprehensive genomic analyses continue to obscure its true contribution to global malaria transmission and epidemiology [11].

In terms of its physiopathology, *P. malariae* generally produces milder clinical manifestations than *P. falciparum* [10,12,13]. However, *P. malariae* infections can occasionally reach parasitemia levels comparable to those of *P. falciparum* and have, in some cases, been associated with severe malaria [4,5,14]. *P. malariae* commonly occurs as part of mixed-species infections rather than as mono-infections and can influence the clinical course and transmission dynamics of other co-infecting *Plasmodium* species [7,8,15]. It also exhibits altered susceptibility to antimalarial compounds [13,16–18]. These factors could explain the persistence of *P. malariae* in regions where *P. falciparum* prevalence is declining [12].

Despite the epidemiological and clinical importance, our knowledge of the basic biology of this neglected parasite remains extremely limited. The development in humans differs from that of other human malaria species by exhibiting a longer intraerythrocytic developmental cycle (IDC) of approximately 72 hours and a remarkable capacity for long-term persistence in the host [12,19]. Unlike *P. vivax*, *P. malariae* does not form dormant hypnozoites in the liver. Instead, chronic low-grade parasitemia may persist for years or even decades, likely due to continuous low-level asexual replication and immune evasion [7,9,10]. The prepatent period has also been shown to vary, ranging from 16 to 59 days depending on the strain [20]. Regarding the sporogonic cycle, eight *Anopheles* species have been reported to transmit this malaria parasite [20], but studies remain very limited and the full range of competent vector species is unknown [21]*.*

The development within the mosquito (i.e., sporogony) is also distinct from *P. falciparum*, which develops for 12–14 days [22,23], while *P. malariae* can take longer, from 14 to 21 days [20].

These distinct human and mosquito-associated parasite phenotypes likely reflect species‑specific genomic features. The publication of the *P. malariae* reference genome revealed that this species has the largest genome of all human-infecting *Plasmodium* which is partly due to the presence of large species-specific families or lineage-specific expansions [13,24,25]. Many of these correspond to multigene families located in telomeric and sub-telomeric regions. Indeed, in *P. malariae*, around 40% of the genome is sub-telomeric [25], compared to ~10% in *P. falciparum* [26]. Examples include the *pir* gene family, present in all human malaria parasites except in *P. falciparum* [27,28], the STP1 family, which is expanded in *P. malariae*, and two novel multigene families organized in tandem termed *fam-m* and *fam-l* (formerly *fam-a*) [25,29]. Interestingly, the *fam-m* and *fam-l* families do not have orthologs in any other species, but they display structural similarity to a *P. falciparum* merozoite surface protein known to be essential for red blood cell invasion, Rh5 [25,30]. Despite the fact that most of these specific families are likely involved in host-parasite interactions, virulence and pathogenesis, their transcriptional dynamics and biological functions in *P. malariae* still remain understudied. Further, it is currently unknown whether these multigenic gene families exhibit clonally variant gene expression, or whether there are additional gene families in *P. malariae* regulated through this mechanism. *P. malariae* also contains a specific repertoire of the ApiAP2 family of transcription factors. Their role and significance for the development and survival of *Plasmodium* are being increasingly recognized, and several ApiAP2 proteins have been described to be master regulators in various life-stage transitions [31–33]. Although early annotations underestimated ApiAP2 conservation because of sequence divergence outside functional domains, recent studies show that this transcription factor family is highly conserved across malaria parasites. However, conserved DNA-binding motifs do not necessarily translate into conserved regulatory networks, as target genes can vary substantially between species. Consequently, the ApiAP2 networks in *P. malariae* remain unknown and their function may differ from those described in *P. falciparum* [25,31,32].

Aside from these well‑known gene families, several *P. malariae* genes remain uncharacterized and lack any functional annotation. An alternative to the hypothesis that new gene functions were acquired in this lineage is that some of the species’ distinctive phenotypic traits may have arisen from differences in the transcriptional regulation of gene sets that are otherwise conserved. This phenomenon has been reported for example when comparing *P. falciparum* and *P. vivax* genes encoding MSP and RBP proteins [34]. However, because of the paucity of transcriptomics and epigenomics data in *P. malariae*, no evidence exists for differences in regulation compared to other species.

Compared to the extensive gene expression studies available for blood stages of *P. falciparum* [35–38] and *P. knowlesi* [39,40], transcriptomic data for *P. malariae* remain limited. These include (1) two single‑cell RNA‑seq datasets for blood‑stage parasites within the Malaria Cell Atlas comprising only ~300 *P. malariae* cells, and (2) a bulk RNA‑seq study that profiled the *P. malariae* transcriptome in the context of an experimental human infection. For the latter, data partly corresponded to the gametocyte stage, but other intraerythrocytic stages were present, and due to the very low amount of mRNA, expression was detected just for ~20% of the genes [41]. This paucity of data is partly attributable to the low parasitemia typically observed in *P. malariae* infections, as well as the current inability to maintain this species in continuous *in vitro* culture.

Throughout the mosquito life cycle, parasite numbers grow exponentially, with hundreds to thousands of forms concentrating first in the midgut and later in the salivary glands [42,43]. These mosquito tissues provide a rich source of parasite material making the mosquito an ideal model system to investigate *P. malariae* transcriptional regulation [44,45]. Transcriptomic data for mosquito stages is available for *P. falciparum*, *P. vivax*, and *P. berghei*. Previous studies have reported just-in-time coordinated waves of gene expression controlling major developmental transitions during sporogony [46–53]. In relation to the AP2 family of TFs in *P. falciparum* and *P. berghei*, ApiAP2-encoding genes display a temporal dynamic in gene expression during sporogony similar to the one reported for the blood cycle, but the TFs are distinct and mosquito stage-specific [54]. A few master regulators have been identified controlling expression cascades of the oocyst and sporozoite stages, chiefly the PfAP2-EXP TF, which has binding sites in the promoters of hundreds of sporozoite-specific genes [50,54,55]. As for multigene families, earlier work in *P. falciparum* has shown that mosquito passage activated the expression of several multigene families with key functions in virulence and pathogenesis in humans but unknown functions in the mosquito host, including *var* genes [56]. Whether these temporal expression patterns are conserved or differ in *P. malariae*, potentially reflecting species-specific differences in virulence, transmission, or vector interactions, remains unknown.

In this study, we aim to characterize the transcriptome of *P. malariae* during its development in the mosquito. To this end, we conducted a series of experimental infections of *An. gambiae* mosquitoes using *P. malariae*-infected blood from human donors and performed RNA-seq of mosquito midguts and salivary glands at 16 and 18 days post-infection (dpi). For the first time, we identified species- and stage-specific transcriptional programs in this pathogen during sporogony, including several genes of unknown function, and unique expression patterns of the ApiAP2 family of regulatory proteins. We also describe the expression of known multigene families implicated in parasite virulence and immune evasion, and genes/families showing evidence of transcriptional heterogeneity in other *Plasmodium spp*.

## Materials and methods

### Mosquito rearing and experimental infections

Three- to 5-day-old female *An. gambiae* s.s. laboratory mosquitoes, sourced from an outbred colony established in 2008 and repeatedly replenished with F1 from wild-caught mosquito females collected in Soumousso, near Bobo-Dioulasso, southwestern Burkina Faso (West Africa), were used for experimental infections. Mosquitoes were maintained under standard insectary conditions (27  ±  2 °C, 80  ±  5% relative humidity, 12:12 LD). The detection of *P. malariae* parasites in the blood of malaria-infected patients was performed by light microscopy. Two independent experimental infections, biological replicates, were carried out by membrane blood feeding in the laboratory as described previously [57,58].

Briefly, females were fed through membranes on *P. malariae* gametocyte-infected blood. The blood samples consisted of whole venous blood from asymptomatic carriers, containing both asexual parasites and mature gametocytes. Thereafter the mosquitoes were given access to a solution of 5% glucose/ 0.05% 4-aminobenzoic acid *ad libitum*. Dissection of infected mosquito midguts and salivary glands was performed in situ at 16 days post-infection (dpi, blood meal) for midguts and salivary glands of infection 1 and 2, and additionally at 18 dpi for salivary glands of infection 2. Mosquito midgut infection was confirmed microscopically. Immediately after dissection, tissues of individual mosquitoes were pooled (10–20 midguts and 30–50 salivary glands), stored in TRIzol (Invitrogen) and frozen at −80 °C until subsequent processing.

### RNA isolation, RNA-seq library preparation and sequencing

We prepared RNA-seq libraries from *P. malariae*-infected midguts and salivary glands obtained in the two independent experimental infections (Table A in S1 Appendix). Total RNA was extracted from pools of midguts and salivary glands using the TRIzol manufacturer protocol. RNA concentration was quantified using a Qubit 2.0 Fluorometer, and RNA integrity was determined with an Agilent 2100 Bioanalyzer. We used the SMART-Seq HT Kit for the synthesis of cDNA from total RNA, and the Nextera XT DNA Library Prep kit (Illumina), for library preparation, following manufacturer’s instructions. Libraries were sequenced using Illumina NovaSeq 6000 using 2x250 bp paired-end reads. The total number of raw reads per sample ranged from ~172 million to 255 million (Table B in S1 Appendix).

### RNA-seq data processing and gene expression analyses

We assessed the sequence quality and rRNA abundance (using GC content as a proxy) using FastQC v0.11.9 [59]. To remove rRNA contamination we used SortMeRNA v4.3.4 [60] with default parameters and the database smr_v4.3_sensitive. If required because of a different number of paired reads after processing, the script repair.sh from the BBMap v38.97 was used [61].

To decontaminate and extract *P. malariae* reads, an initial taxonomic classification analysis was performed using KrakenUniq v0.6 [62] for pathogen identification [63]. We used default parameters and the pre-built index kuniq_standard_plus_eupath_minus.kdb.20200816 to include the databases archaea, bacteria, viral, human, UniVec_Core, and EuPathDB. To extract only the reads assigned to *P. malariae*, we used KrakenTools v1.0.1 [63] with default parameters, except for -t 5858. The resulting reads underwent a second round of classification against a custom database of *P. malariae* and various *Anopheles* reference genomes (PlasmoDB v68 and VectorBase-59, respectively) [64–66].

The resulting RNA-seq reads were mapped against the *P. malariae* UG01 reference genome using HISAT2 v2.2.1 with default parameters except for --very-sensitive [67]. Quality control was performed with Qualimap v2.2.2d [68] and SAMtools (v1.12) [69]. We extracted paired unique reads with samtools view -q 60 -f 0x2 and removed sequencing duplicates using picard MarkDuplicates v2.24.0 [70] with default parameters except for --REMOVE_SEQUENCING_DUPLICATES true. To ensure proper alignment and remove noise, we also filtered out alignments shorter than 100 bp by analysing the CIGAR string. After all filtering above, the number of reads mapping to the *P. malariae* genome (PmUG01) varied between ~109k and ~723k per sample, with uniquely mapped reads representing 76–99% of these (Table B in S1 Appendix). Mean mapping quality scores were ~35, and overall alignment rates to the *P. malariae* reference genome were 78–100%. Correlation and PCA plots were performed by the deepTools2 (v3.5.1) [71] functions multiBamSummary, plotCorrelation, and plotPCA.

After the alignment and quality control steps, raw and TPM-normalized counts at the gene level were obtained using CoCo (v.0.2.6) [72]. Default parameters were used except for determining strandedness (-s 0) and providing the insert size average for each sample (-i). The *P. malariae* annotation was adapted beforehand using basic functions, to add the biotype to the gene entries and to extend their 5’ ends by 1.5 Kbp so 5’UTRs were accounted for. Genes with < 25 TPM counts when summing all samples were excluded. We categorized normalized counts in high, medium or low groups dividing the signal in three quantile groups using the *Hmisc::cut2* R function [73].

To assess the presence of other *Plasmodium* species in the samples (Table C in S1 Appendix), a competitive mapping approach was implemented using BWA-MEM v0.7.19 [74]. To do this, a combined reference was generated by concatenating the genomes of *P. malariae, P. falciparum*, both species of *P. ovale (P.o. curtisi and P.o. wallikeri*), *P. vivax*, and the mosquito *Anopheles gambiae*. After alignment, the same filtering protocol as in the previous analysis was rigorously applied: sequencing duplicates were removed with Picard, reads with (-q 60 -f 0x2) were selected, and alignments shorter than 100 bp were discarded using CIGAR string.

We assessed the potential multiclonality of the infections by SNP calling using BCFtools v1.14 [75]. We used bcftools mpileup and bcftools call with default parameters except for -cv -Ov --ploidy 1, we discarded indels, applied a quality threshold of 30 with VCFtools v0.1.16 [76], and filtered keeping only high quality genotypes (DP > 5), positions covered by more than five reads were retained to ensure reliable allele calls. Allele frequencies were manually computed using the DP4 field from the VCF files, which records the number of reads supporting: DP4 [1] = forward reads for the reference allele, DP4 [2] = reverse reads for the reference allele, DP4 [3] = forward reads for the alternate allele, and DP4 [4] = reverse reads for the alternate allele. The total depth for the reference and alternate alleles was calculated as ref_total = DP4 [1] + DP4 [2] and alt_total = DP4 [3] + DP4 [4], respectively, and the allele frequency (AF) was computed as:


$$\begin{array}{c} AF=\frac{\text{alt}\_\text{total}}{\text{ref}\_\text{total }+\text{ alt}\_\text{total}} \end{array}$$

This approach allows accurate estimation of allele frequencies at positions with sufficient read coverage.

To assess the presence of mixed parasite developmental stages in the midgut and salivary gland samples, we first integrated our data with that of other *Plasmodium* species using the corrplot function and Pearson correlation. Alternatively, we performed digital deconvolution using the CIBERSORTx webserver [77,78]**.** Signature matrices were built using *P. falciparum* and *P. berghei* scRNA-seq data from the Malaria Cell Atlas initiative [79,80] and cell fractions in each bulk sample (Table D in S1 Appendix) were imputed using 1:1 orthologs between species. Default parameters were used except for batch correction with S-mode using the same scRNA-seq reference matrix, quantile normalization enabled, and 500 permutations performed.

Differential gene expression analyses were conducted using the DESeq2 R package v1.30.0 [81]. Correlation and PCA plots by deepTools2 showed higher similarity between stages than between infections (S1A–S1B Fig). Accordingly, experimental infections were used as replicates and included as a covariate in the formula. The significance threshold was established at p.adj (adjusted P-value) < 0.05. Gene Ontology (GO) enrichment analyses were performed by PlasmoDB tools (default parameters, P-value threshold 0.05). Unless otherwise specified, we used R for basic statistical and data mining analyses [82] and the packages iheatmapr (v0.5.0) [83] and ggplot2 (v3.3.3) [84] for plotting results. The genome browser used to assess genome-wide transcriptional profiles, motif instances, and gene features was IGV [85]. Coverage profiles were converted to bigwig files normalized by bins per million mapped reads (BPM) using deepTools2 [71] bamCoverage function.

### Identification of putative novel multi-copy variable gene families in *P. malariae*

We aimed to identify *P. malariae* genes encoding protein products with similar or identical functions, which could serve as a proxy for expecting some degree of heterogeneity in their transcriptional patterns. Using PlasmoDB v68 annotation, we focused on the ‘product description’ information for all *P. malariae* genes and extracted those with identical values. Subsequently, genes with very generic descriptions, such as ‘hypothetical protein,’ ‘conserved *Plasmodium* protein, unknown function,’ ‘conserved protein, unknown function,’ and genes belonging to multigene families previously described by other authors [25,56] were eliminated. This elimination was methodologically necessary to prevent the artifactual clustering of hundreds of unrelated genes sharing the same generic placeholder text, which would have compromised the subsequent manual tandem-location analysis and sequence-homology validation. Next, a manual search was performed leveraging PlasmoDB visualization to determine whether genes sharing a description were also located in tandem. Finally, beyond the semantic similarity of their annotated product, we assessed the degree of sequence homology between all the members of the gene families. To this end, we first extracted the nucleotide sequences via PlasmoDB interface and used blastn v2.16.0 [86] with default parameters except for -evalue 0.0001. To establish a reference threshold, we computed the intra-family homology of the families of well-described multi-copy and clonally variant gene families, namely *fam-l*, *fam-m*, *pir*, *stp1*, *clag*, *rbp*, *trag*. We report ~80% as the average identity in these families and, together with an identical description of the coding product, an identity above this threshold was set as a requirement to be considered as a multigene family candidate in this study.

As an alternative strategy, we analyzed external single-cell RNA-seq data to identify *P. falciparum* and *P. berghei* genes showing evidence of cell-to-cell transcriptional heterogeneity in mosquito stages. These were herein named hypervariable genes (HVGs). Our assumption was that the ortholog genes in *P. malariae*, if available, could be HVGs also in *P. malariae* and show a pattern of transcriptional variability during sporogony. To identify HVG candidates for each *Plasmodium* species and developmental stage independently, we extracted for each gene the range of reported expressions in all cells, except for outliers (following the 1.5*IQR rule) to compensate for possible dropout events. We categorized all genes with a non-zero range (i.e., non-equal maximum and minimum non-outlier values, so the genes are expressed with variability in multiple cells) into Very High, High, Medium, Low, and Very Low categories (Hmisc::cut2 with g = 5 parameter instead of the g = 3 above). We consider the genes categorized as Very High/High to be those showing larger cell-to-cell variability of expression. Next, we used the function FindVariableFeatures in the Seurat R package v5.1.0 [87] with default parameters except for selection.method = “vst” mean.cutoff = c(0.1, 8), dispersion.cutoff = c(1, Inf). As for the parameter “nfeatures”, which is required and determines the arbitrary number of variable features to be obtained based on a standardized variance, we used the number of genes in the Very High/High categories above. We considered high-confidence HVG candidates the genes that are both in the Very High/High groups and in the result of the FindVariableFeatures, and investigated the transcriptional profiles of ortholog genes in *P. malariae,* or any.

### Genes lacking functional annotation in *P. malariae*

Genes annotated as ‘hypothetical protein’, ‘conserved Plasmodium protein, unknown function’, or ‘conserved protein, unknown function’ were classified as lacking functional annotation. To infer potential functions, we first retrieved any available GO terms for these genes from PlasmoDB v68. For genes without GO annotations, we identified orthologs in *P. falciparum, P. berghei, and P. vivax* using the orthology relationships curated in PlasmoDB v68 (OrthoMCL), and examined whether GO terms were annotated for the corresponding orthologs in those species. Ultimately, for the genes without known evidence of function but expressed in our dataset, we applied the PANNZER (Protein ANNotation with Z-scoRE) webserver to obtain automated predictions of protein functions and GO annotations [88,89]. The annotations were performed using default parameters, i.e., considering a cut-off value of PPV (Positive Predictive Value) ≥ 0.3, and using the same parameters applied to trypanosomatids in a recent study [90].

### Comparative analysis of AP2 orthologs and motif enrichment between *Plasmodium malariae* and other species

Automated clustering algorithms, such as the OrthoMCL method implemented in VEuPathDB/PlasmoDB, are known to have limited accuracy when applied to highly divergent protein families, including the ApiAP2 transcription factors. Consequently, for this family, orthologous relationships were inferred using OrthologR [91], since sequence conservation is largely restricted to the AP2 DNA-binding domain rather than extending across the full-length proteins. Protein sequences corresponding to the ApiAP2 repertoire were used as input for similarity searches against reference proteomes of *P. falciparum* and *P. vivax* (PlasmoDB v68)*.*

Similarity searches were performed using OrthologR, applying an e-value threshold of 1e-3. This relatively permissive threshold was selected to retain homologous sequences in the ApiAP2 family and avoid the loss of biologically relevant matches. Orthologs were identified using a Best Hit (BH) approach, whereby, for each *P. malariae* protein, the top-scoring hit in the target species was retained. To validate our results, orthology assignments were compared against the curated annotations and orthology assignments reported in the *P. malariae* UG01 reference genome published by *Rutledge et al.* (2017) [25].

We performed *de novo* motif analysis using HOMER v4.11 [92] on the promoters (1 Kbp upstream) of different gene sets of interest, such as those highly expressed (at least one infection has high expression), to identify the binding sites for *P. malariae* ApiAP2 transcription factors that are orthologous to *P. falciparum* AP2 proteins using the previously published database of validated *P. falciparum* ApiAP2 motifs [31]. We used the *findMotifsGenome.pl* module with the parameters “-len 8,10,12 -size given -mset all -dumpFasta” and as reference with the parameter “-mcheck” the *P. falciparum* AP2 motif library file. We also conducted motif scan and located the occurrence of motifs using the *annotatePeaks.pl* module from HOMER. Motif selection was based on strong statistical enrichment, indicated by a very low P-value (or FDR), high occurrence in target sequences, and low occurrence in background sequences. Motifs showing the strongest correspondence to known *P. falciparum* ApiAP2 binding motifs (named primary) were prioritized.

### External data

We reanalyzed the RNA-seq bulk dataset available for *P. malariae* blood stages (*i.e.*, mixed stages but correlated with gametocytes) [41] following the same methods applied for mosquito samples in this study.

We downloaded the available scRNA-seq data, corresponding to 13 cells, for *P. malariae* blood stages in the Malaria Cell Atlas [80]. We manually computed TPM counts using the function calculateTPM from the scuttle R package v1.6.3 [93] and reported mean values.

We downloaded from PlasmoDB v68 already published TPM-normalized counts for *P. falciparum* [49,50,94] and *P. berghei* [95–97] mosquito stages. A dataset of *P. vivax*-infected *Anopheles stephensi* tissues is available [47], but original authors only addressed the mosquito responses and did not analyze parasite sequences. To obtain TPM-normalized counts for *P. vivax* in mosquito midguts and salivary glands, we reanalyzed this data following the same methods applied for *P. malariae* samples in this study, except for using the *P. vivax Sal-1* reference genome [98,99].

We downloaded scRNA-seq reference data used in the analyses above from the Malaria Cell Atlas, corresponding to *P. falciparum* and *P. berghei* [79,80,100]. We leveraged the existing annotation. In *P. falciparum*, we used the available cell labels “ookinete” (112 cells), “sporozoite-oocyst” (158 cells), and “sporozoite-salivary glands” (257 cells), while in *P. berghei* we used the available cell labels “ookinete” (141 cells), “oocyst” (252 cells), and “sporozoite-salivary glands” (153 cells).

In all cases above, ortholog genes between *P. malariae* and other *Plasmodium* species were determined using PlasmoDB v68 and OrthoMCL [101], and only syntenic genes were used except for ApiAP2 family (see above).

## Results

### The mosquito as an experimental host for studying *P. malariae* infections

RNA-seq libraries were produced from adult female *Anopheles gambiae* s.s. mosquitoes fed with *P. malariae*-infected blood obtained from human volunteers from a malaria-endemic area in Burkina Faso, Africa (see Methods). Two independent experimental infections were performed; two samples are available for midguts and three for salivary glands (Fig 1A; Table A in S1 Appendix). The replicability analysis of the RNA-seq data revealed higher similarity and clustering by stages rather than by experimental infection, which supports the use of the infections as biological replicates in our transcriptomics analyses (S1A–S1B Fig).

**Fig 1:**
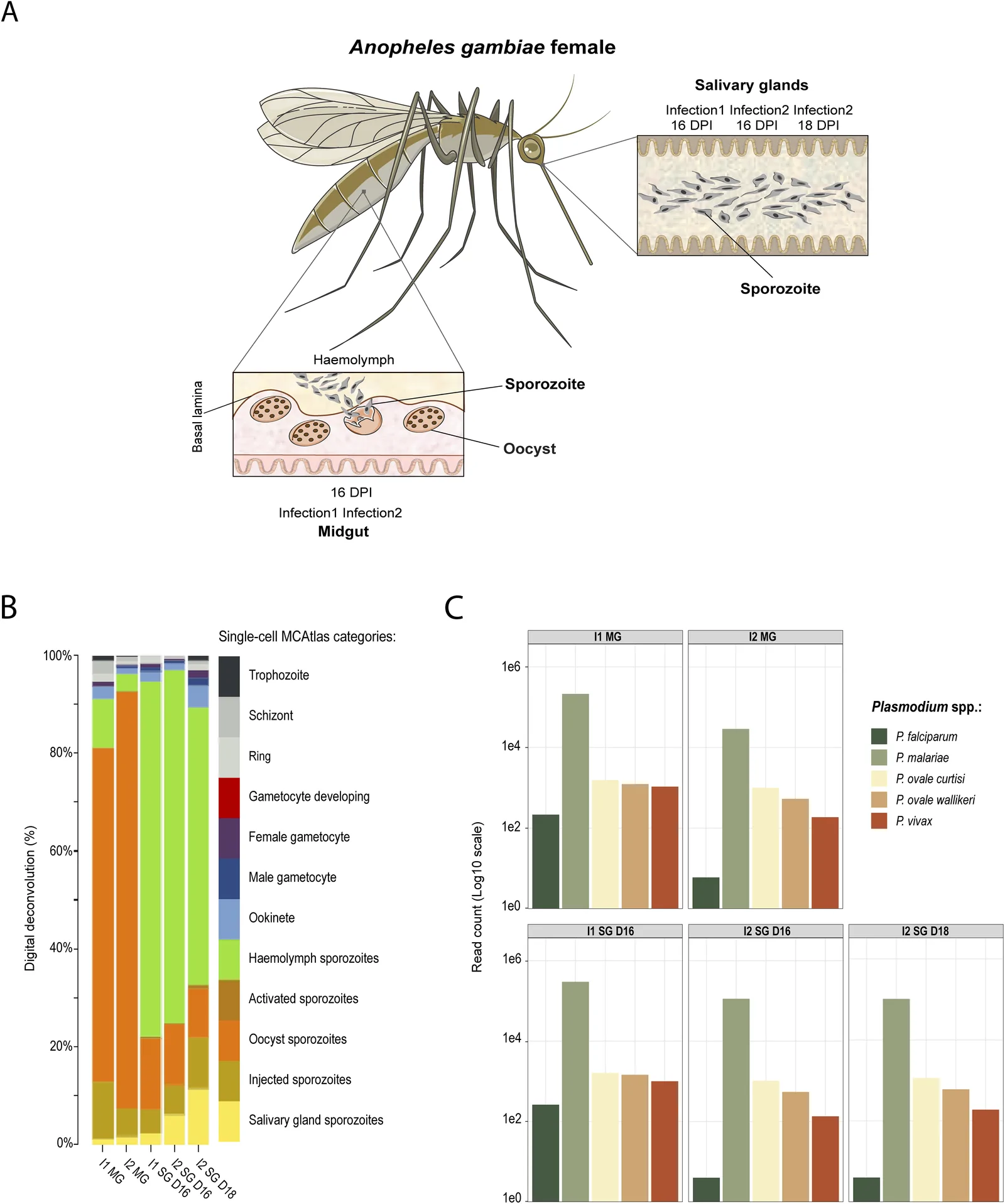
The mosquito as an experimental host to study *Plasmodium malariae* infection. **(A)** Schematic representation of *Plasmodium malariae* development in its natural vector *Anopheles gambiae*. After ingestion of *P. malariae* infected-blood, gametocytes differentiate into gametes in the midgut, and fertilise to form a zygote that in 24h differentiates into an ookinete that traverses the mosquito midgut epithelium where oocyst formation occurs. Between 14–21 days post infection, the precise timing is uncertain in *P. malariae*, mature oocysts release sporozoites into the haemocoel (haemolymph), from where they migrate to the salivary glands. The sporozoites invade the salivary glands, becoming available for transmission to the vertebrate host during a subsequent mosquito bite. The samples analysed in this study correspond to the different tissues colonized by the parasite during its development in the vector, that is, mosquito midgut and salivary glands containing oocysts and immature sporozoites, and sporozoite stages, respectively. Panel A contains icons by Servier [102], used and modified under a CC BY 4.0 licence. The original images were modified by the authors. **(B)** Barplot showing the deconvolution of the *P. malariae* bulk RNA-seq data for midgut and salivary gland samples using previously published *P. falciparum* single-cell data (Table D in S1 Appendix) [79,80]. Oocyst and sporozoite stages account for the larger proportions. **(C)** Barplots representing the distribution of read numbers (Log10 scale) for five human-infecting Plasmodium species (*P. falciparum, P. malariae, P. ovale curtisi, P. ovale wallikeri,* and *P. vivax*). A consistent overrepresentation of *P. malariae* is observed in all samples (Table C in S1 Appendix).

To use the mosquito as an experimental host, a series of pre-analytical steps and considerations were made. First, we had to assume some uncertainty in relation to the precise developmental stage of *P. malariae* parasites isolated from the midgut of infected mosquitoes. That is, human malaria parasites typically display a variable development period in the mosquito midgut (between 7–14 days). It is after 14–21 days that the oocyst eventually bursts, releasing sporozoites into the hemolymph that travel to the salivary glands [20]. Therefore, achieving precise isolation of the different developmental stages in the infected midguts is challenging [77,103]. Further, the development of *P. malariae* in the mosquito remains poorly characterized compared to other species, so caution is warranted. Due to this uncertainty and the slower extrinsic incubation period of this species, in this study infected midguts were dissected at 16 (dpi) whereas salivary glands were dissected at 16- and 18-dpi. For these two time points at least one of the stages is expected to be present in either tissue: midgut 16 dpi = oocysts + immature sporozoites, and salivary gland 16–18 dpi = mature sporozoites.

In this context, accurate developmental staging is intrinsically difficult, as infected midguts are likely to contain a mixture of mature oocysts and immature sporozoites, even when samples are collected at identical post-infection time points. We compared our *P. malariae* RNA-seq data with time-course data sets from *P. falciparum*, *P. vivax* and *P. berghei*, and looked for significant correlation (Pearson’s, P < 0.001). For the midgut samples, in *P. falciparum* we reported highest correlation coefficients against immature sporozoites in midguts (~50%), followed by ookinetes (~25%) and oocysts (~15%), whereas in *P. vivax* the peaks of correlation corresponded to early oocysts (~37%), and in *P. berghei* to ookinetes (~30%) (S1C Fig). The peaks of correlation for the salivary gland samples coincided with mature sporozoite datasets in all cases (~75% correlation) (S1C Fig). To further validate the assumption that *P. malariae* reads in midguts mostly captured immature sporozoites in oocysts, we leveraged the *P. falciparum* and *P. berghei* single-cell RNA-seq data (available at the project Malaria Cell Atlas [79,80]) to perform digital deconvolution of our *P. malariae* RNA-seq bulk data of mosquito stages [77]. We found that immature sporozoites were predicted to make up the largest proportion of cells in the midgut samples (mean ~77% of “sporozoite (oocyst)” in *P. falciparum* and ~42% of “sporozoite (salivary gland)” in *P. berghei*; Figs 1B; S1D; Table D in S1 Appendix). Accordingly, the largest fractions in salivary gland samples were also classified as sporozoites (Figs 1B; S1D; Table D in S1 Appendix). All this considered, we assumed that our salivary glands samples, either at day 16 or 18 dpi, captured the transcriptome of *P. malariae* sporozoites, while midguts appeared to contain a mixed composition of oocysts and sporozoite stages (named “oocyst/sporozoites” hereinafter).

Second, to explore the possibility that our samples contain mixed parasite infections, a competitive mapping analysis was performed (see Methods). The results revealed an overrepresentation of *P. malariae* reads in all samples (Fig 1C, Table C in S1 Appendix), representing between 94.4% (midguts I2) and 98.6% (salivary glands I1) of the total parasite reads. In all samples, other species such as *P. ovale curtisi* and *P. ovale wallikeri* were detected but in a much lower proportion (not exceeding 3.3% and 1.7%, respectively) whereas *P. falciparum* and *P. vivax* were detected at negligible levels (<0.6%). Because of the high sequence conservation across *Plasmodium* genomes, these trace amounts likely represent competitive mapping artifacts rather than genuine mixed infections.

A final consideration of this work, given that we dealt with natural infections, was the potential presence of multiple clones or genotypes [104]. To rule out any confounding patterns, we inferred the frequency of homozygous/heterozygous SNPs in our RNA-seq data (see Methods section). Results indicate that despite different SNPs numbers detected, the majority of them were homozygous (>90% of SNPs with allele frequency parameter, AF = 1), suggesting that the infections in this study were monoclonal (S2A Fig).

### Uniqueness and dynamics of the transcriptome of *P. malariae* during sporogony

The analysis of *P. malariae* RNA-seq libraries resulted in the detection of 3,699 genes that we considered to be expressed (genes with > 25 TPM, see Methods). These represent ~55% of the reference transcriptome (6,715 genes). A total of 2,586 out of the 3,699 expressed genes corresponded to protein coding genes that we categorized as highly expressed (see Methods) in at least one of the stages in any infection (Table E in S1 Appendix). Amongst the top expressed genes in *P. malariae* mosquito-stages appear orthologs of well-characterized sporozoite markers from *P. falciparum* and *P. berghei*, such as SPECT1 (sporozoite microneme protein essential for cell traversal) and CSP (circumsporozoite protein) (Figs 2A and S3A, respectively). Other highly expressed genes include gene families specific to *P. malariae*, such as MSP3 or the gamete antigens 27/25 (Table E in S1 Appendix).

**Fig 2:**
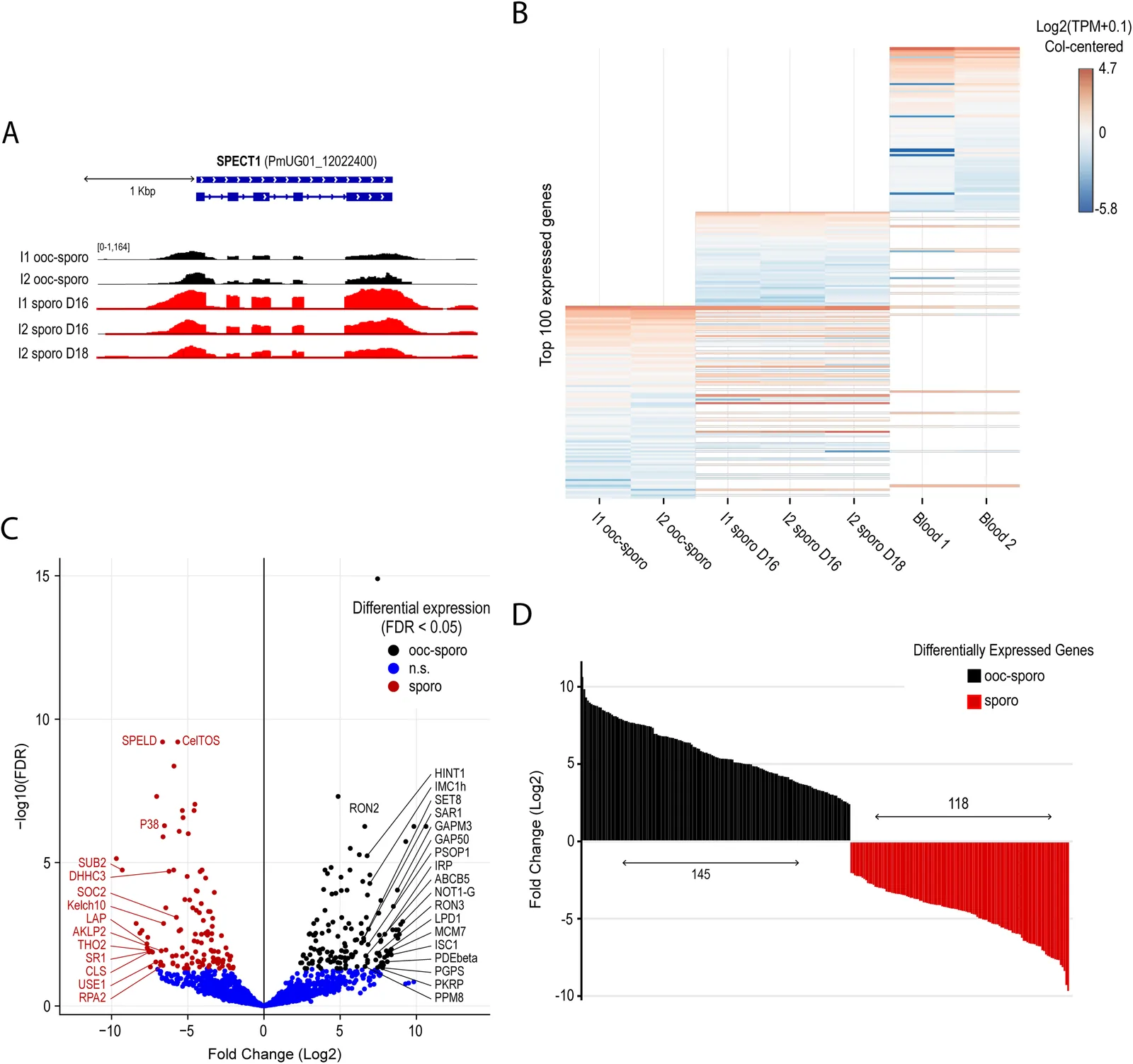
Stage-specific transcriptional profiles of *P. malariae* during sporogony. **(A)** Gene expression profiles in the region containing the gene encoding the SPECT1 protein (PmUG01_12022400). The tracks shown are for midgut oocysts/sporozoites (black) and salivary gland sporozoites (red). All tracks are shown at equal scale. **(B)** Heatmap for the top 100 most highly expressed genes (Log2(TPM+0.1) col-centered) in different stages: oocysts/sporozoites (I1 ooc-sporo D16, I2 ooc-sporo), sporozoites (I1 sporo D16, I2 sporo D16, I2 sporo D18), and blood (Blood 1, Blood 2). For stages where no expression of certain genes is detected, the color is blank. **(C)** Volcano plot displaying the magnitude of the change and the statistical significance for differentially expressed genes (DEGs) between *P. malariae* developmental stages. The x axis is the fold change difference between stages, and the y axis shows the level of significance. Significant DEGs are marked in black and red for midgut and salivary gland samples, respectively, the blue ones are not significant. A positive fold change (x axis) indicates higher expression in midgut oocysts/sporozoites, while a negative value indicates the opposite trend (higher gene expression in salivary gland sporozoites). **(D)** Histogram showing the number of differentially expressed genes (DEGs) in midgut oocysts/sporozoites (ooc-sporo) and salivary gland sporozoites (sporo).

When comparing *P. malariae* blood and mosquito stages, most genes appear to be unique to a particular stage of development as shown in Fig 2B where most of the top 100 more expressed genes in mosquito stages did not appear as expressed in the blood stages. Between midgut oocyst/sporozoites and salivary gland sporozoites, we identify 2,043 developmentally regulated genes with a |log2 Fold Change| > 2 and up to 10.6 (Fig 2C, Table E in S1 Appendix). However, for most of them the change did not reach statistical significance in the differential analyses. That is, only 263 could be considered as significant differentially expressed genes (DEGs): 145 more expressed in midgut samples (oocysts/sporozoites) and 118 in salivary gland sporozoites (Fig 2D; Table E in S1 Appendix).

Our results partially overlap with the dynamic transcriptome during sporogony described in other *Plasmodium spp.* [54,105]. For example, DEGs overexpressed in midgut oocysts/sporozoites include inner membrane complex proteins (IMC1a, 1c, 1e, IMC20), the rhoptry protein (RON12), glideosome proteins (GAPM3, GAP50, GAPM2, GAPM1), RALP, TREP, secreted ookinete protein 1 (PSOP1), apical sushi protein ASP, or histones (H2A, H2B, H4) (Table E in S1 Appendix). Amongst DEGs overexpressed in salivary gland sporozoites, we found the apical membrane antigen AMA1, TRAMP (thrombospondin-related apical membrane protein), SPATR (secreted protein with altered thrombospondin repeat domain), SLARP (sporozoite and liver stage asparagine-rich protein), TLP (trap-like protein), 6 cysteine proteins (P38, P52, P36, B9, PmUG01_03014600), PmUG01_07044000 (profilin motility protein), PUF2 (mRNA binding protein, inhibitor and controls sporozoite conversion to liver stages), PLP1, PLP2 (perforin for host traversal), serpentine receptor SR1, GAMA, TRAP (thrombospondin-related anonymous protein), CelTOS (cell traversal protein for ookinetes and sporozoites), GEST (gamete egress and sporozoite traversal), or SPELD (sporozoite protein essential for cell traversal) (Table E in S1 Appendix). Similarly, the GO overrepresentation analyses performed on midgut oocyst/sporozoite- and salivary gland sporozoite-specific genes, showed enriched terms corresponding to functional activities that conform with the expectations for each stage of development (Table F in S1 Appendix). These include biosynthetic processes or metabolism in midgut oocysts/sporozoites, and gliding, motility, response to stress or multi-organism symbiotic processes in salivary gland sporozoites (S2B–S2C Fig; Table F in S1 Appendix).

Based on the orthology annotations available in PlasmoDB v68, 1,889 *P. malariae* genes lacked assigned orthologs in *P. falciparum, P. vivax, or P. berghei*, of which 309 were expressed in our dataset (Table E in S1 Appendix). Thus, genes without assigned orthologs represented approximately 16% of the mosquito-stage transcriptome and 5% of the 6,715 genes in the *P. malariae* genome. These estimates are dependent on the orthology assignments available in PlasmoDB v68 and may change as genome assemblies, gene annotations, and orthology relationships are refined in future database releases. Of the 309 expressed genes without assigned orthologs, 32 were differentially expressed, with 19 showing higher expression in midguts and 13 in salivary glands. These represented ~2% of the 1,889 genes without assigned orthologs and ~12% of the total mosquito-stage DEG repertoire (Table E in S1 Appendix). Among them were genes assigned to known gene families, including a 6-cysteine protein (PmUG01_03014600), merozoite surface protein 3 (PmUG01_06022200), an early transcribed membrane protein (PmUG01_10012700), a putative knob-associated histidine-rich protein (PmUG01_14085700), and a Plasmodium-interspersed repeat (PIR) protein (PmUG01_13059300), as well as numerous genes annotated as ‘hypothetical protein’ or ‘conserved Plasmodium protein, unknown function’.

GO enrichment analysis of the 309 *P. malariae*-specific genes revealed enrichment of terms related to transcriptional regulation, including ‘regulation of DNA-templated transcription’. At the molecular function level, ‘sequence-specific DNA binding’ and ‘transcription regulator activity’ were significantly enriched after multiple-testing correction. In the cellular component category, terms associated with ‘host cellular components’ and ‘other organism parts’ were significantly overrepresented, together with membrane-associated components (Table G in S1 Appendix).

Next, we looked at genes without annotation (no gene description) in the *P. malariae* reference genome (PlasmoDB v68, see Methods) which represent ~20% of the entire genome (1,331 genes). Of these, we report in our dataset the expression of 1,056 genes (~80%); and 91 out of the 1,056 are differentially expressed between midgut oocysts/sporozoites and salivary gland sporozoites in this study (Table H in S1 Appendix). To further investigate their possible function, we first considered genes without annotated gene description in *P. malariae* but with GO terms (347 out of the 1,056 genes), GO enrichment analysis revealed significant enrichment of membrane-associated cellular components, while terms related to DNA damage and integrity checkpoints showed nominal enrichment but did not remain significant after multiple-testing correction (Table I1 in S1 Appendix, S3B Fig). For the remaining 709 *P. malariae* genes without an associated GO term, we investigated whether they have annotated GO terms in other *Plasmodium* species. To this end, we looked at their ortholog and syntenic genes in *P. falciparum*, *P. vivax* and *P. berghei*. We found 1,449 orthologs for 555 *P. malariae* genes, and of these 263 orthologs corresponding to 201 *P. malariae* genes have associated GO terms in other species. Enrichment analysis for GO cellular components (CC) terms revealed an overrepresentation of kinetochore and centromeric chromosome regions in *P. vivax* and *P. berghei*, with additional enrichment of membrane-associated components in *P. berghei*. In *P. falciparum*, enriched cellular components included kinetochore and centromeric chromosome regions and the apicoplast/plastid (Table I2 in S1 Appendix).

Finally, we applied PANNZER2 [90] to the complete set of 1,056 expressed genes lacking functional annotation to predict new gene functions. This analysis generated annotations for 213 genes, including 48 genes that previously had no associated GO terms. To assess the reliability of these predictions, we compared the PANNZER2-derived GO terms with the computed and manually curated GO annotations available for a subset of genes. Among the 165 genes for which both annotation types were available, 79 showed matching annotations, corresponding to a 47.9% agreement rate (Table H in S1 Appendix). The newly inferred GO terms are provided in Table H in S1 Appendix. Overrepresentation analysis leveraging the newly assigned function of the previously unannotated genes did not identify significantly enriched GO terms.

Altogether, our results suggest that the *P. malariae* transcriptome during sporogony broadly conforms to the just-in-time model described in other Plasmodium species, while also displaying distinct features. Notably, the gene repertoire expressed during mosquito-stage development includes several species-specific genes, some of which remain uncharacterized and may play roles in parasite adaptation and interactions with both mosquito and human hosts.

### Master regulators of the *P. malariae* transcriptome during sporogony

The transcription factors from the ApiAP2 family have been shown to act as master regulators of developmental transitions in *Plasmodium spp*., but evidence about their function and essentiality is available only in few reference species, including *P. falciparum*, *P. vivax*, and *P. berghei* [32,33]. Here, we used the mosquito experimental model to find out more about the expression dynamics and target genes of these transcriptional regulatory proteins in *P. malariae*. To comprehensively assess the evolutionary conservation of the *P. malariae* ApiAP2 family, we performed a systematic orthology analysis against *P. falciparum* and *P. vivax* (see Methods). We found that 28 of the 29 *P. malariae* ApiAP2 factors have orthologs in *P. falciparum*, while the remaining gene is conserved in *P. vivax* but not in *P. falciparum* (Table J1 in S1 Appendix). These assignments differ from those reported in PlasmoDB v68 (22 ApiAP2 factors with orthologs), likely reflecting the limitations of automated clustering approaches for ApiAP2 proteins, which are highly divergent outside their conserved DNA-binding domains. Overall, our results are largely consistent with those of *Rutledge et al.* (2017) [25], with 26 of 29 *P. falciparum* orthology assignments and all *P. vivax* assignments in agreement. The few discrepancies were limited to highly divergent genes, for which orthology is difficult to determine (Table J1 in S1 Appendix) [25]. Three genes showed differences between the analyses. First, we identified PF3D7_1222400 as the *P. falciparum* ortholog of PmUG01_10026800, whereas *Rutledge et al.* did not report an ortholog. Second, *Rutledge et al.* assigned PF3D7_0622900 as the ortholog of PmUG01_11040700, but we found no corresponding *P. falciparum* ortholog. Finally, for PmUG01_14033900, our best-hit approach identified PF3D7_1007700 as the ortholog, while *Rutledge et al.* assigned PF3D7_1317200. These differences may reflect the distinct criteria used for orthologue identification. In these cases, the assignments reported by *Rutledge et al.* were manually curated and complementary supported by conserved synteny.

We find that the majority of *P. malariae* AP2 TFs (23 out of 29) are expressed during sporogony, although only a few AP2 TFs show noticeable peaks of expression that are consistent across infections (Table E in S1 Appendix). In the midgut oocysts/sporozoites, the top 10 expressed AP2 TFs are in this order: AP2-O4 (PmUG01_12014000), AP2-HC (PmUG01_12060900), AP2-EXP (PmUG01_12050500), AP2-O, AP2-I(PmUG01_09052200), AP2-G3 (PmUG01_14033900), AP2TEL (PmUG01_11040700), AP2-L (PmUG01_02024900), PmUG01_09024900, PmUG01_09017400, PmUG01_08023500. In salivary gland sporozoites, the most expressed are in this order: PmUG01_05037900, AP2-EXP (PmUG01_12050500), AP2-P (PmUG01_09017400), AP2-I (PmUG01_08023500), AP2-L (PmUG01_02024900), AP2-O (PmUG01_09052200), AP2-O5 (PmUG01_12067500), AP2-O4 (PmUG01_12014000), PmUG01_09024900 (Fig 3A, Table E in S1 Appendix). Remarkably, *P. malariae* AP2-EXP (PmUG01_12050500) is an example of highly expressed AP2 in both midgut oocysts/sporozoites and salivary gland sporozoites (Table E in S1 Appendix). The ortholog *P. falciparum* AP2-EXP has been reported to be highly expressed in mosquito stages and potentially involved in transcriptional regulation during sporogony [50]. Similarly, PmUG01_09024900, whose ortholog in *P. falciparum* (PF3D7_1115500) is currently of unknown function, appears to be more expressed in mosquito-stages according to published data [49,50].

**Fig 3:**
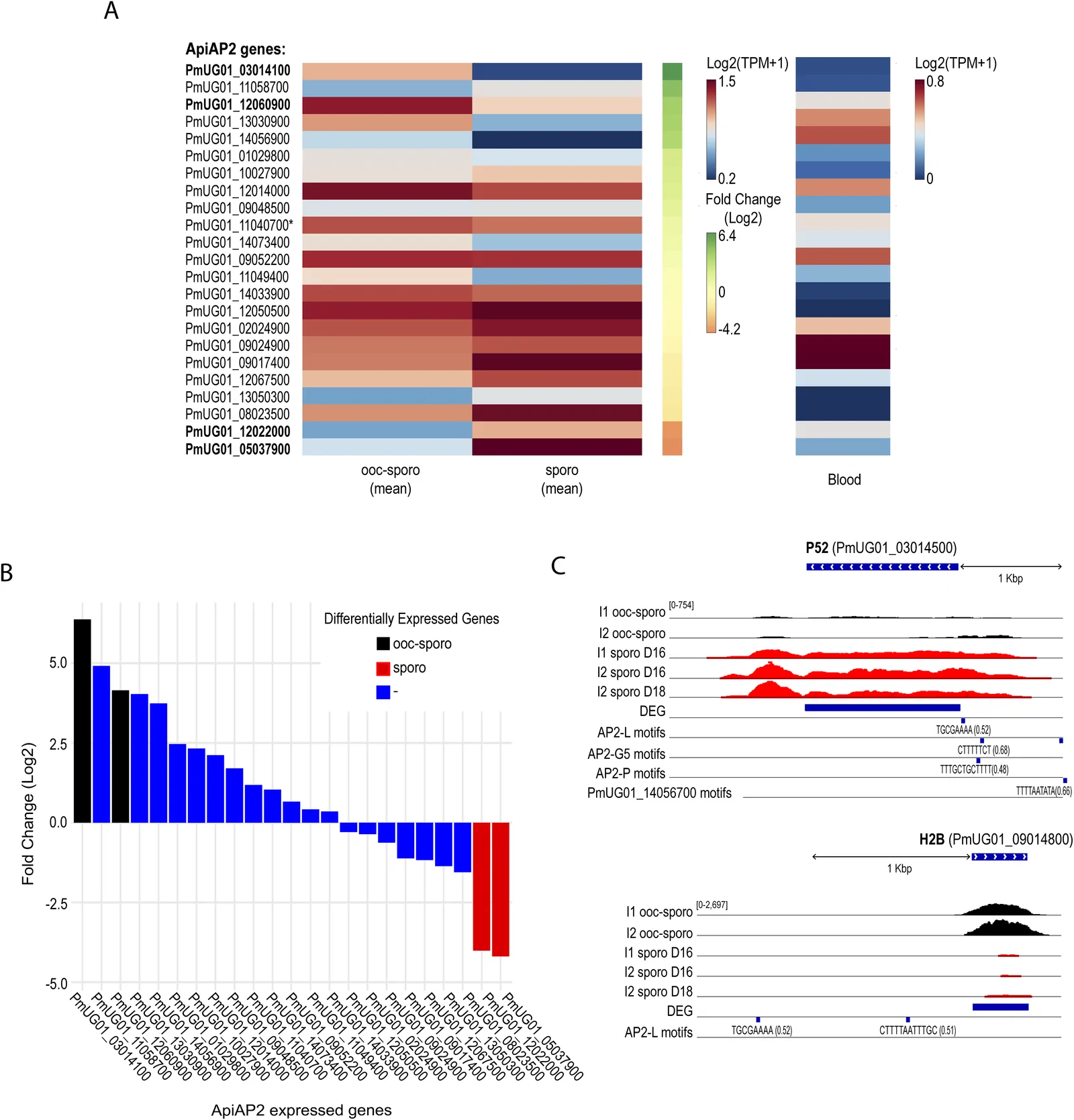
Expression profiles of the AP2 family of TFs in *P. malariae.* **(A)** Heatmap of log2-transformed TPM expression levels for all 23 AP2 expressed genes across blood and mosquito stages (midgut oocysts/sporozoites D16, salivary gland sporozoites D16/D18). Expression values were column-scaled, highlighting relative expression across stages rather than absolute differences. Differentially expressed genes (DEGs), determined using DESeq2 with adjusted FDR < 0.05, are shown in bold. Genes without orthologs in *P. falciparum* (*) are indicated. **(B)** Differential expression of AP2 TFs genes in *P. malariae* mosquito stages. Bars highlight the magnitude of expression differences representing the fold change (log2) between midgut oocysts/sporozoites (black) and salivary gland sporozoites (red), DEGs (padj < 0.05). PmUG01_11058700 was not classified as a DEG despite a large fold change due to high inter-replicate variability, including a near-zero value in one biological replicate, which reduced statistical significance. **(C)** Genomic browser visualization (IGV) of RNA-seq coverage at loci potentially regulated by differentially expressed AP2 TFs (containing P52 and histone H2B encoding genes), together with the locations of binding motifs for AP2-L, AP2-G5, AP2-P and PmUG01_14056700. The tracks shown are for midgut oocysts/sporozoites (black) and salivary gland sporozoites (red) for infections 1–2. All tracks are shown at equal scale.

Four *P. malariae* AP2-TFs appear differentially expressed in the mosquito cycle: AP2-SP2 (PmUG01_03014100, ortholog to *Pf*AP2-SP2, PF3D7_0404100 and *Pv*AP2-SP2, PVP01_0303400), AP2-HC (PmUG01_12060900*,* ortholog to *Pf*AP2-HC, PF3D7_1456000 and *Pv*AP2-HC*,* PVP01_1250900), AP2-HS (PmUG01_12022000, ortholog to *Pf*AP2-HS, PF3D7_1342900 and *Pv*AP2-HS, PVP01_1211900), and PmUG01_05037900 (ortholog to PF3D7_0420300 and PVP01_0529800) (Fig 3B; Tables E, J1 in S1 Appendix). The transcriptomics profile for ApiAP2 genes in *P. malariae* mosquito stages contrasts with the pattern previously reported by *Woodford et al*. in blood stages (of mixed composition, but correlation peaking against gametocytes) [41]. By reanalyzing that data (see Methods), we found that the AP2 TFs AP2-P (PmUG01_09017400) and PmUG01_09024900 displayed comparable peaks of expression, while gene expression levels of the rest of the AP2-TFs reported in the mosquito stages were very low in blood stages (S4A Fig; Table E in S1 Appendix). Looking at *P. malariae* single-cell data of mixed blood stages available in the first Malaria Cell Atlas [80] (13 cells, mostly late rings and trophozoites), the TFs more expressed are AP2-O2 (PmUG01_10027900), AP2-LT (PmUG01_01029800), or PmUG01_14073400, with many others being expressed at lower levels (S4B Fig). Overall, these developmental differences between the transcriptomes further support the stage-specificity of the *P. malariae* ApiAP2 family and point to a few mosquito-specific AP2-TFs playing a more prominent role during sporogony.

To study the regulatory networks of *P. malariae* AP2 TFs during infection in the mosquito, including putative binding sites and target genes, we conducted a DNA-binding motif enrichment analysis on the upstream regions (i.e., potential promoter / 5’UTR regions) for the set of genes that we categorized as highly expressed (1,005 genes, see Methods) (Table E in S1 Appendix). For this analysis, we used as a reference *P. falciparum* AP2 TF binding sites from previous *in silico* analyses [31], but we focused on the AP2 genes that are orthologous between *P. malariae* and *P. falciparum* (Table J1 in S1 Appendix). From this analysis, we obtained 41 motifs matching AP2 genes, of these 23 are primary and 18 are secondary motifs (Table J2 in S1 Appendix). Considering only primary motifs, the most enriched motif, TGCGAAAA, was associated with PfAP2-L (ortholog: PmUG01_02024900), followed by three PfAP2-G5-associated motifs (ortholog: PmUG01_09048500): CAAAATAAAGCA, AACGAACAAACA, and CTTTTTCT. Additional enriched motifs included TTTGCTGCTTTT (PfAP2-P; PmUG01_09017400), TTTTAATATA (PF3D7_1222400; PmUG01_14056700), and CGTTTACG (PfAP2-G; PmUG01_14056900).

We screened the upstream regions of the 1,005 highly expressed genes for the enriched ApiAP2 motifs identified above (Table J3–J7 in S1 Appendix). AP2-L motifs were detected in 623 genes, including 142 differentially expressed genes (DEGs). AP2-G5 exhibited the highest number of genes with at least one motif (850), of which 186 were DEGs. Motifs associated with PF3D7_1222400, AP2-P, and AP2-G were identified in 462, 592, and 455 genes, respectively, corresponding to 103, 125, and 99 DEGs (Table J3–J7 in S1 Appendix). GO enrichment analysis of genes targeted by the five AP2 transcription factors identified above revealed BP terms associated with pathogenicity, including biological processes involved in host cell interaction, motility, and egress. Enriched molecular functions (MF) terms were dominated by catalytic activity, protein kinase signalling, and transcriptional regulation, while enriched cellular component (CC) terms showed strong localisation to the apical complex and other invasion-related organelles. Examination of motif occurrences revealed potential combinatorial regulation by multiple ApiAP2 factors, particularly among invasion-related genes. For example, P52 contained binding sites for PmAP2-L, PmAP2-G5, PmAP2-P, and PmUG01_14056700 (Fig 3C; Table J3–J7 in S1 Appendix), whereas CSP was associated only with PmAP2-L, PmAP2-G5 and PmAP2-G (S3A Fig; Table J3–J7 in S1 Appendix). P38 harboured motifs for PmAP2-L, PmAP2-G5, and PmAP2-G (S4C Fig; Table J3–J7 in S1 Appendix), while ELC contained binding sites for PmAP2-L, PmAP2-G5, PmAP2-P, and PmAP2-G (S4D Fig; Table J3–J7 in S1 Appendix). Among chromatin-related genes, H2B was identified as a potential PmAP2-L target (Fig 3C; Table J3–J6 in S1 Appendix), whereas H3.3 contained motifs for PmAP2-L, PmAP2-P, and PmUG01_14056700 (S5A Fig; Table J3–J6 in S1 Appendix). Collectively, these findings support coordinated ApiAP2-mediated regulation of key *P. malariae* genes across its life cycle in the mosquito.

### *P. malariae* transcriptionally variant gene families expressed in the mosquito

*Plasmodium* species possess a diverse number of gene families that are highly polymorphic and transcriptionally variable, named clonally variant gene (CVG) families in which a single gene can display alternative transcriptional states independently of the genotype and the stage of development. Some of these families and display mutually exclusive expression. Multigene families are key to parasite survival and are involved in a variety of processes including red blood cells invasion, metabolism, transmission, and transcriptional regulation [106–108]. Gene expression patterns and dynamics of multigene and CVG families remain mostly unknown for *P. malariae* [109].

We first examined gene expression patterns of known clonally variant and multigene families as described by others [25,56], in *P. malariae* mosquito stages. Out of 1,222 genes belonging to 13 families, 123 genes appear expressed in mosquito stages in this study, 10% of the total. These included *fam-m* (13/283 genes), *fam-l* (20/396 genes), *pir* (11/257), *stp1* (16/159), *6-cys* (10/13), *acs* (3/4), *clag* (2/4), *etramp* (6/7), *fikk* (17/20), *lpl* (1/3), *phist* (12/29), *rbp/rh* (2/5), *trag* (10/42) (Table K in S1 Appendix). Additionally, 7 out of the 123 expressed genes are DEGs, being in all cases more expressed in salivary gland sporozoites, of which 5 belong to the *6-cys* family and 2 to the *fikk* family (Table K in S1 Appendix). Of these 123 genes, 57 belong to the category of highly expressed in at least one stage: 27 for midgut oocyst/sporozoites and 46 in salivary gland sporozoites, 16 in both stages. 17 genes were categorized as medium expression (all of them in salivary gland sporozoites). Comparing the list of known multigene families expressed in mosquitoes (123 genes) with the *P. malariae* blood-stage transcriptome, we obtained a total of 58 matches [41] (Table K in S1 Appendix). Of these, 5 genes are also differentially expressed in this study: 2 *fikk* encoding genes and 3 genes of the *6-cys* family (Fig 4A; Table K in S1 Appendix). Finally, 18 *P. malariae* multigene family members, including 7 genes belonging to the 6-cys family, 2 etramp and 9 serine/threonine kinase contain motifs associated to AP2-L and/or AP2-G5, AP2-P, PmUG01_14056700, AP2-G (Table J3–J7 in S1 Appendix).

**Fig 4:**
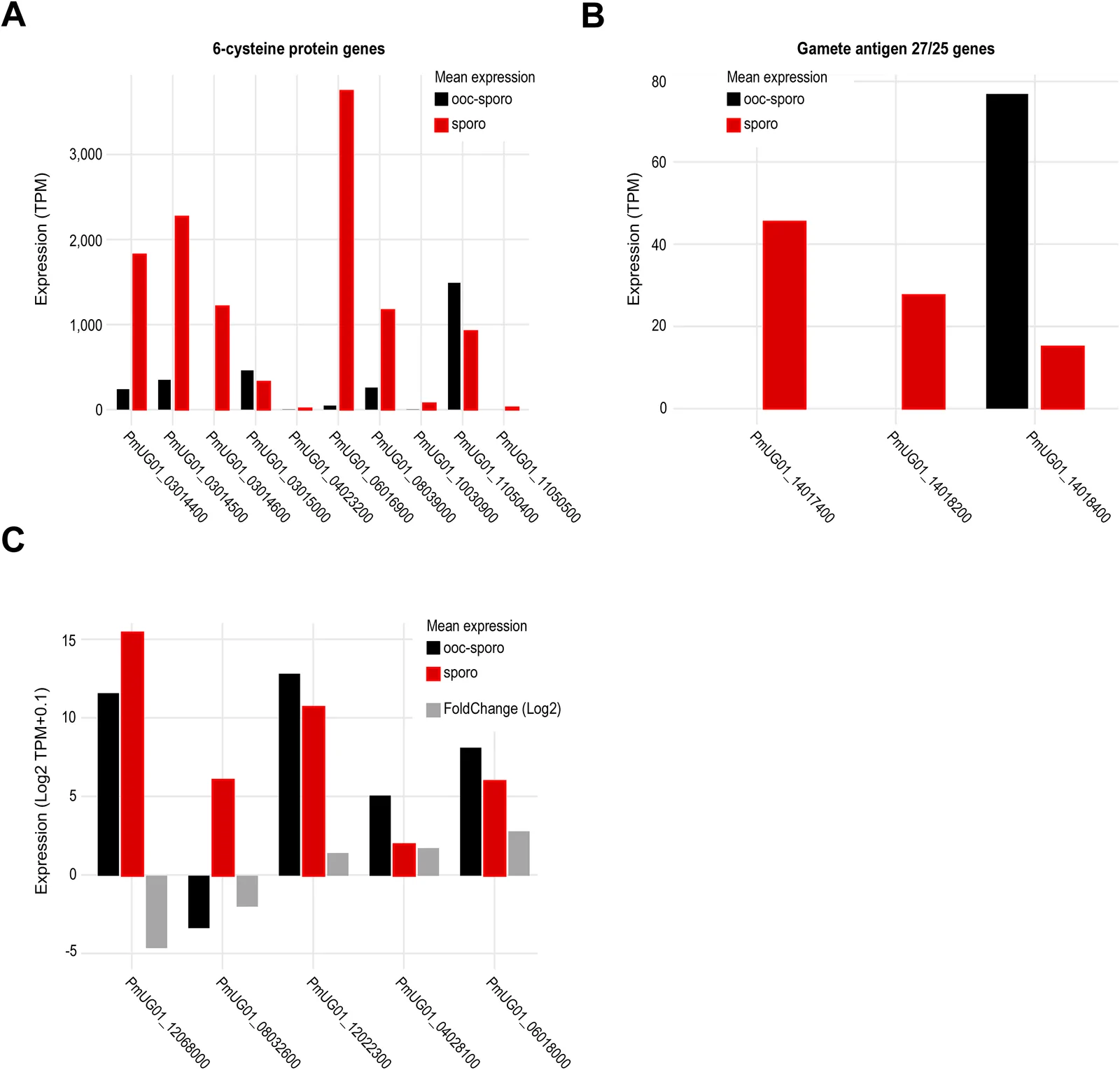
*P*. malari*ae* multigene families expressed in mosquito stages. **(A**) Barplots showing gene expression levels for ten members of the multigene family *6-cys* expressed in *P. malariae* mosquito stages. **(B)** Barplots showing gene expression levels for the three members of the family gamete antigen 27/25 described as potential novel multigene family in *P. malariae*. **(C)** Barplots showing for 5 random hypervariable genes (HVGs) computed based on *P. falciparum* scRNA-seq data the gene expression levels in *P. malariae* midgut oocyst/sporozoites and salivary gland sporozoites samples, and the difference (log2FC).

Secondly, we aimed to identify potentially new and/or unexplored multigene families in *P. malariae* using a homology-based strategy. For this, we first searched for genes that: 1) encode apparently the same product that can or cannot be located in tandem (i.e., close to each other) and 2) show a sequence homology between gene copies that is analogous to that of the known families (~80%, see Methods). Our homology-based strategy revealed 1,396 *P. malariae* genes deemed to be potentially part of multigene families. Of these, 324 genes displayed at least one blast hit with identity >80% with any other member of its alleged multigene family, and the vast majority of them were “Plasmodium_exported_protein”, a big family of 522 members. Out of the high confidence list of multigene candidates mentioned before (324 genes), 32 genes are expressed in *P. malariae* mosquito stages, out of which 10 are highly expressed (2 in midgut oocyst/sporozoites and 8 in salivary gland sporozoites) and 6 display ortholog genes in other *Plasmodium* species, pointing to species-specific functions (Table K in S1 Appendix). Of the 32 candidate genes, 13 were expressed in both mosquito and blood stages. Although several genes exhibited stage-specific differences in expression, none were identified as significantly differentially expressed, likely reflecting variability among biological replicates. The list of multigene candidates expressed in *P. malariae* mosquito stages includes members of the families *Msp7-like* proteins, gamete antigens 27/25, and serine repeat antigens (Table K in S1 Appendix). More specifically, we detected that most genes coding for the gamete antigen 27/25 proteins display a higher expression in salivary gland sporozoites, except for the PmUG01_14018400 gene, whose expression is higher in midgut oocysts/sporozoites (Fig 4B). As for the serine-repeat antigen genes, we identified different stage-specificity; some genes were predominantly expressed in midgut stages (PmUG01_04024400, PmUG01_04024800, PmUG01_04025200) and others with higher expression in the sporozoite stage (PmUG01_04024600, PmUG01_04024700).

Thirdly, to further expand the list of candidate multigene families in *P. malariae*, we analysed mosquito stages single-cell data available at the Malaria Cell Atlas for *P. falciparum* (S5B Fig) *and P. berghei* [79,80]. Specifically, we identified genes showing patterns of cell-to-cell transcriptional heterogeneity that have ortholog/syntenic genes in *P. malariae*. Following the nomenclature by the Malaria Cell Atlas, these genes were herein considered hypervariable genes (HVGs) candidates. We applied a high-confidence approach in which only genes showing a large diverse range of expression or being identified by an approach based on standardized variance (see Methods), were considered for subsequent analysis. We analyzed independently single-cell data for *P. falciparum* developmental stages (including ookinetes, midgut oocysts and salivary gland sporozoites, see Methods) and identified a total of 484 putative HVGs candidates with an ortholog in *P. malariae* and expressed in mosquito stages in this study (Fig 4C; Table K in S1 Appendix). Of these, 60 genes are differentially expressed in the mosquito (31 DEGs more expressed in midgut oocysts/sporozoites and 29 DEGs in salivary gland sporozoites). We confirmed that the majority of the 484 HVGs are highly expressed genes (87 highly expressed in oocyst/sporozoites, 94 highly expressed in sporozoites and 171 in both stages). These include the malaria vaccine antigen CelTOS, the Puf-family RNA-binding protein Puf2, and the pore-forming protein EXP2, merozoite TRAP family proteins: PmUG01_12028900, PmUG01_02019600 and the gamete egress sporozoite traversal (GEST) protein. From single-cell data of independent *P. berghei* developmental stages (i.e., ookinetes, oocysts, and sporozoites stages) we captured 1,482 HVGs candidates with ortholog and syntenic genes in *P. malariae* that are expressed in mosquito stages in this study. Of those, 144 genes appear developmentally regulated (79 DEGs more expressed in midgut oocysts/sporozoites and 65 DEGs more expressed in salivary gland sporozoites). Out of the 1,482 HVGs, 1,138 genes are highly expressed in our data. Several relevant examples include inner membrane complex proteins (IMC1c, IMC1e, IMC1k, ALV7, ISC1), HSP20, PSOP1, PIMMS22, glideosome (GAPM1, GAPM2, GAPM3), SPATR, TRAP, GEST, P38, SPELD, CelTOS, profilin, RON2, IMP2, PPM5, PPM8, CLAMP, P36, B9, HECT1, histone(H2B), AMA1, GAMA, SSP3. Finally, a total number of 91 and 879 HVGs candidates (from *P. falciparum* and *P. berghei*, respectively) appear expressed both in blood and mosquito stages.

Altogether, the analysis of multigene and hypervariable gene families described in other *Plasmodium spp*., or proposed as such in *P. malariae* based on the comparative analysis above, resulted in a list of hundreds of putative transcriptionally variant genes that are medium or highly expressed in *P. malariae* mosquito stages. A fraction of them are differentially expressed. Whether they actually can display clonally variant expression during *P. malariae* sporogony and which are their roles in virulence and pathogenesis, remain to be investigated in future studies.

## Discussion

Many aspects of the biology of the human malaria parasite *P. malariae* remain mostly unknown [109]. This study represents the first genomic study of this neglected pathogen during sporogony in the mosquito, with the objective of understanding the uniqueness of its transcriptome in terms of species-specific gene repertoires, regulatory factors and virulence families having a role during this critical part of the malaria parasite life cycle.

Given the inability to continuously culture *P. malariae* in vitro, mosquitoes provide an ideal experimental host in which parasite development and transmission can be studied under natural infection conditions [19]. Although our results are restricted to mosquito stages and may not be generalisable to other stages of the parasite life cycle, given the scarcity of transcriptomic data for *P. malariae*, characterising sporogonic development provides valuable insights into gene function and parasite biology and represents an important first step towards understanding this understudied species.

Experimental transmission studies of *P. malariae* remain scarce because low parasitaemia and gametocyte densities make mosquito infections difficult to establish [110]. In addition, *P. malariae* infections are relatively uncommon and frequently occur as co-infections with *P. falciparum* and *P. ovale*. Despite these challenges, we successfully established two experimental *P. malariae* infections in *Anopheles gambiae*. Although the limited number of biological replicates reduces statistical power, the novelty of these data provides an important foundation for understanding the biology of this neglected parasite.

An additional challenge is the uncertainty surrounding the duration of the extrinsic incubation period in *P. malariae*. We therefore selected an intermediate sampling point of 16 dpi to maximise parasite recovery from both midguts and salivary glands. This approach carries the risk of capturing a mixed population of mature oocysts and immature sporozoites even selecting the same post-infection time points. Digital deconvolution of the bulk RNA-seq data showed that the largest fraction of reads in the midgut corresponded to oocyst-derived immature sporozoites. Such a mixed transcriptomic profile is expected given the asynchronous progression of parasite development in the mosquito.

Another aspect to consider is the use of *Anopheles gambiae s.s.* for experimental infections. Although *An. gambiae* is capable of supporting *P. malariae* development and transmission under laboratory conditions [110], its role as a natural vector remains poorly understood. Field studies indicate that *P. malariae* can infect multiple *Anopheles* species, including *An. gambiae s.s.*, and that the dominant vector likely varies geographically [111–113].

A further consideration is that malaria-infected individuals in endemic areas often harbour multiple *Plasmodium* species and parasite genotypes. Although mosquitoes were infected with *P. malariae*-positive blood, our samples consisted predominantly of *P. malariae* reads, with only minor contributions from *P. ovale* (1.7%-3.3%) and *P. falciparum* (0.5%), both of which commonly co-occur with *P. malariae* in Burkina Faso [114]. Moreover, analyses of within-sample genetic diversity indicated that infections were predominantly monoclonal, supported by the high proportion of homozygous SNPs (>90%) detected in the *P. malariae* genome.

We identified expression of 3,699 genes during *P. malariae* sporogony, representing approximately 55% of the 6,715 annotated genes in the genome. This transcriptome coverage is notable given the low parasitemia typical of *P. malariae* infections and the limited parasite material available from mosquito stages. It also substantially expands previous *P. malariae* transcriptomic data, which, as acknowledged by the authors, was limited to 1,463 blood-stage and gametocyte genes due to low RNA yield [41]. Although direct comparisons across *Plasmodium* species are constrained by methodological differences [115], the number of expressed genes is comparable to that reported for sporozoites of other species (~3,500–4,200 genes) [116]. Most expressed genes with orthologs in other *Plasmodium* spp. were associated with functions expected during sporogony, including metabolism and biosynthesis in oocysts and motility-related processes in sporozoites [54,116,117]. The stage-specific transcriptional profiles observed during sporogony support the “just-in-time” model described in other malaria parasites, whereby genes are expressed shortly before their products are required [34,54,118]. An example consistent with this pattern is PIMMS22, whose orthologs are essential for oocyst development in *P. falciparum* and *P. berghei* and which we report to be differentially expressed in *P. malariae* [119]. Similarly, sporozoites showed high expression of *AMA1*, a key protein involved in motility and host-cell invasion and an important malaria vaccine target [120].

Differential expression analysis identified 263 genes (7%) as developmentally regulated, whilst a further 1,780 genes showed >4-fold expression (|log2FC| > 2) differences between stages without reaching statistical significance. This likely reflects the limited number of biological replicates and the inherent variability of *P. malariae* clinical isolates, including differences in parasite genotypes, host blood characteristics, and asynchronous development within mosquito midguts.

The mosquito-stage transcriptome included a substantial number of genes with no assigned orthologs in other major Plasmodium species. Based on PlasmoDB v68 orthology annotations, 309 expressed genes initially appeared to lack orthologs in *P. falciparum*, *P. vivax*, and *P. berghei*. However, subsequent OrthologR analysis identified orthologs for seven ApiAP2 genes, reducing the number of genes without assigned orthologs to 302. These genes represent approximately 8% of the transcriptome detected during mosquito-stage development. This estimate should be interpreted cautiously, as orthology assignments depend on the current quality of genome assemblies and annotations and may change as these resources improve. Moreover, some genes belonged to established *Plasmodium* gene families, for which orthology relationships can be difficult to resolve because of extensive sequence divergence or lineage-specific expansion. In contrast, numerous genes were annotated only as ‘hypothetical protein’ or ‘conserved Plasmodium protein, unknown function’, making them particularly attractive candidates for investigating P. malariae-specific biology and host-associated adaptations.

Despite recent improvements in *P. malariae* genome annotation [25], approximately 20% of genes remain functionally uncharacterized. We detected expression of 1,056 such genes without annotation, including 91 differentially expressed genes. By integrating GO annotations from *P. malariae* and orthologous genes in other *Plasmodium* species, and by applying PANNZER2-based annotation, we assigned putative functions to a subset of these genes, including 48 that previously lacked GO annotations. Nevertheless, no significant GO enrichment emerged from the newly annotated gene set, underscoring the substantial gaps that remain in our understanding of *P. malariae* biology while providing a framework for future functional studies.

ApiAP2 transcription factors are key regulators of gene expression throughout the *Plasmodium* life cycle [31,32,94]. While only two ApiAP2 genes have previously been reported to be expressed in *P. malariae* blood stages [41], we detected transcription of 23 of the 29 ApiAP2 family members during sporogony. All 23 expressed ApiAP2 genes have orthologs in either *P. falciparum* or *P. vivax* based on our analysis (21 applying the method by *Rutledge et al.*). Expression patterns were highly stage-specific, with 14 genes enriched in oocysts/midgut sporozoites and nine in salivary gland sporozoites, although only four reached statistical significance for differential expression. In addition to expected mosquito-stage regulators such as AP2-O, AP2-O5, and AP2-G [121,122], several expressed factors are orthologous to regulators of liver-stage or blood-stage development in *P. falciparum,* suggesting possible functional divergence in *P. malariae*, including AP2-L, AP2-I, SIP2, and AP2-EXP [33,123–125]. Motif analysis of AP2-L, AP2-G5, AP2-P, PmUG01_14056700 and AP2-G identified numerous putative target genes, including many differentially expressed genes and key sporozoite factors involved in motility and host-cell invasion such as *CSP*, *P52*, *P38*, and *ELC* [126–128]. Shared targets among multiple ApiAP2 proteins, including the histone variants *H2B* and *H3.3*, further suggest combinatorial regulation [129,130]. Overall, these findings support a conserved but complex ApiAP2 regulatory network in *P. malariae* mosquito stages, while highlighting potential differences from other *Plasmodium* species that warrant further functional validation.

Multigene families play key roles in immune evasion, host-cell invasion, and parasite development [56]. Unlike *P. falciparum*, *P. malariae* lacks the *var* gene family and has a reduced *pir* repertoire, but shows expansions of other multigene families, including *stp1*, *fam-l*, and *fam-m* [19,25,29,56]. The functions and expression patterns of these families remain poorly understood. Despite the challenges of analysing highly repetitive subtelomeric genes, we detected expression of 123 multigene family members during sporogony, including *fam-l*, *fam-m*, *pir*, *stp1*, tryptophan-rich antigens, and *6-cys* proteins. Only seven were significantly differentially expressed between stages, most belonging to the *6-cys* family. Notably, proteins such as P36, P52, P38, P41, and PSOP12 were highly expressed in sporozoites, consistent with functions in host-cell invasion and parasite transmission described in other *Plasmodium* species [131–133]. Moreover, some genes from multigene families contained predicted AP2 binding motifs, suggesting regulation by mosquito-stage transcriptional networks.

In contrast to previous blood-stage transcriptomic studies, which detected little or no expression of *fam-l, fam-m,* and *pir* genes [41], these families were actively expressed during mosquito-stage development. We also identified 324 candidate multicopy genes, including serine-repeat antigens, *msp7*-like genes, and members of the expanded gamete antigen 27/25 family. Many were expressed in both mosquito and blood stages, indicating potential roles throughout the parasite life cycle. Using published single-cell datasets from other *Plasmodium* species, we further identified 1,689 candidate hypervariable genes with *P. malariae* orthologs (484 from *P. falciparum* and 1,482 from *P. berghei*, with 277 shared between both species). Of these, 1,271 genes (75%, 73% and 77% of *P. falciparum* and *P. berghei*, respectively) were highly expressed during sporogony and 165 were DEGs. Several candidates, including *CelTOS*, *TRAP*, and *GEST*, are known transmission-stage factors involved in cell traversal, invasion, and parasite development [54,134,135].

Together, these findings reveal a diverse repertoire of multigene and hypervariable genes expressed during *P. malariae* sporogony and highlight candidate multigene families that may contribute to transmission, persistence, and adaptation. However, analyses based on natural infections and short-read sequencing are limited in their ability to resolve highly polymorphic subtelomeric regions. Long-read genome assemblies and single-cell transcriptomic approaches will be required to fully characterize these gene families and determine whether they undergo mutually exclusive expression.

In conclusion, we report for the first time the transcriptome of *P. malariae* during sporogony in the mosquito, filling an important gap related to our current knowledge on the biology of this neglected human malaria parasite. We characterized stage-specific transcriptional programs that recapitulate the different biological functions for each developmental stage during sporogony and conform to what has been described for other malaria parasite species. However, our results also highlight the expression of species-specific gene repertoires during sporogony, including ApiAP2 transcription factors, known *P. malariae* multigene families, and candidate hypervariable and multigene families. Together, these genes may contribute to the unique developmental, transmission, and persistence strategies that distinguish *P. malariae* from other human malaria parasites.

## Supporting information

S1 Fig **(A)** Correlation plot showing similarity between infections (I1, I2) and developmental stages (*P. malariae* oocysts and sporozoites). Samples are ordered by an unsupervised hierarchical clustering approach and clustered by stage rather than biological replicate (experimental infection) both immediately after alignment to reference genome (left) and after processing and computing read counts (right). **(B)** Principal Component Analysis on the biological replicates (I1, I2) and developmental stages (midgut oocysts/sporozoites and salivary gland sporozoites). The plots show the percentage of variance explained by PC1 and PC2 axes and the distribution of the samples along the first two Principal Components. Samples clustered by stage rather than biological replicate. **(C)** Correlation plot showing the similarity between the *P. malariae* samples in this study and previously published transcriptomes during sporogony for *P. falciparum* [49,50], *P. vivax* [47], and *P. berghei* from *PlasmoDB v68* [65]. Expression data are TPM-normalized gene counts. The pairs with significant correlation coefficients are indicated. The peaks of correlation between matching developmental stages in the different species are highlighted in red. **(D)** Barplots showing the deconvolution of the *P. malariae* bulk RNA-seq data samples in this study using previously published *P. berghei* single-cell data [80]. Matching stages account for the larger proportions.
(TIF)

S2 Fig **(A)** Barplots showing the number of SNPs in the sequences when comparing with the reference genome. More than 90% of SNPs have an allele frequency (AF) of 1.00, indicating homozygosity and supporting monoclonality. **(B)** GO terms obtained in the enrichment analysis performed on the 118 differentially expressed genes (DEGs) that appear more expressed in salivary gland sporozoites. **(C)** GO terms obtained in the enrichment analysis performed on the 145 differentially expressed genes (DEGs) that appear more expressed in midgut oocyst/sporozoites.
(TIF)

S3 Fig **(A)** Genomic visualization of highly expressed gene CSP together with the positions of binding motifs for AP2-L, AP2-G5, AP2-P, PmUG01_14056700 and AP2-G. **(B)** Word cloud showing GO enrichment for the 347 “No function” genes with orthologs in *P. malariae* (based PlasmoDB v68)
(TIF)

S4 Fig **(A)** Expression patterns of *P. malariae* AP2 TFs in blood stages, data from *Woodford et al.* [41]. **(B)** Expression patterns of *P. malariae* AP2 TFs taking the average expression of the 13 cells from *P. malariae* single cell RNA-seq data (Malaria Cell Atlas). **(C-D)** Genomic visualization of expressed AP2-regulated loci. Examples of genomic regions include differentially and highly expressed genes P38 (C) and ELC (D), together with the positions of binding motifs for AP2-L, AP2-G5, AP2-P, PmUG01_14056700 and AP2-G. The tracks shown are for midgut oocysts/sporozoites (black) and salivary gland sporozoites (red) for infections 1–2. All tracks are shown at equal scale.
(TIF)

S5 Fig **(A)** Genomic visualization of expressed AP2-regulated loci. Examples of genomic regions include highly expressed gene H3.3, together with the positions of binding motifs for AP2-L, AP2-G5, AP2-P, PmUG01_14056700 and AP2-G. The tracks shown are for midgut oocysts/sporozoites (black) and salivary gland sporozoites (red) for infections 1–2. All tracks are shown at equal scale. **(B)** Boxplots showing the variable expression of five randomly selected hypervariable genes (HVGs) from *P. falciparum* single-cell RNA-seq data (Malaria Cell Atlas). Values represent the reported expression in cells labeled as ookinete, sporozoite (oocyst), and sporozoite (salivary gland).
(TIF)

S1 Appendix Table A. Mosquito experimental infections. Two experimental infections (I1-I2) were performed. Microscopic confirmation of midgut infection including: gametocytemia, prevalence (percentage of infected mosquitoes), intensity of infection (mean number of oocysts), numbers of mosquitoes and the range of oocyst numbers. **Table B.** Read alignments statistics. Summary of RNA-seq alignment and quality control statistics. **Table C.** Summary read counts and percentage distribution for *Plasmodium* species**.** Table representing the number of reads identified in our samples, number of reads are referred to paired end reads. Percentages in parentheses indicate the relative abundance of each species within the total reads. **Table D.** Bulk RNA-seq deconvolution. **(1)** Relative cell-type composition of the *P. malariae* samples in this study based on available *P. falciparum* single-cell RNA-seq profiles [79,80]. **(2)** Relative cell-type composition of the *P. malariae* samples in this study based on available *P. berghei* single-cell RNA-seq profiles from the Malaria Cell Atlas [79,80]. **Table E.** RNA-seq gene expression levels. Gene expression (CoCo-derived TPM-normalized counts) for the *P. malariae* transcriptome. Columns include expression rank (high, medium, or low), functional annotation, differential expression betweendevelopmental stages, orthology assignments from PlasmoDB v68, and gametocyte-stage expression levels obtained through reanalysis of previously published data [41]. **Table F.** Gene ontology terms overrepresentation analyses of the stage-specific differentially expressed genes. Results include the biological processes (BP), molecular functions (MF) and cellular components (CC) GO terms, and the KEGG and MetaCyc metabolic pathways used by PlasmoDB [65]. **Table G.** Gene ontology terms overrepresentation analysis of genes without orthologs in *P. falciparum, P. berghei* and *P. vivax*. Results include the biological processes (BP), molecular functions (MF) and cellular components (CC) GO terms used by PlasmoDB [65]. **Table H.** List of expressed genes lacking a product description (“no function”) based on PlasmoDB v68. Genes may occur in multiple rows, reflecting their representation across orthologs or organisms. Gene expression values are TPM-normalized RNA-seq counts. Columns correspond to functional annotation, orthology information, and GO terms obtained from PlasmoDB [65] or PANNZER2 [88]. **Table I.** Gene ontology terms overrepresentation analysis of *P. malariae* expressed genes without annotated functions based on PlasmoDB v68. Results include GO BP, MF and CC terms for expressed genes **(1)** and GO BP for *P. falciparum, P. vivax* and *P. berghei* ortholog genes **(2). Table J.** AP2 ortholog assignment and motif occurrences in mosquito stages highly expressed *P. malariae* genes. **(1)** Orthology predictions for 29 *P. malariae* AP2 genes (Pm_Gene_ID) against *P. falciparum* (Pf) and *P. vivax* (Pv) are shown. Columns show the orthologs assigned via OrthologR using BH method alongside the reference annotations from *Rutledge et al.* (2017) orthology data, as well as the nomenclature and their primary biological function, validated *in vivo* or *in* *vitro*. Genes with no described function are categorised as putative transcription factors. Note that six AP2 genes listed in Table J1 in S1 Appendix (AP2-Z, AP2-EXP2, and four unnamed AP2 genes) are absent from Table E because they were not detected as expressed in the analyzed samples. **(2)** List of enriched consensus motifs corresponding to known ApiAP2 TFs in the promoters of highly expressed genes in mosquito stages. **(3)** Instances corresponding to AP2-L motifs located at the promoters of highly expressed genes. **(4)** Instances corresponding to AP2-G5 motifs located at the promoters of highly expressed genes. **(5)** Instances corresponding to AP2-P motifs obtained in this study that are located at the promoters of highly expressed genes. **(6)** Instances corresponding to PmUG01_14056700 motifs obtained in this study that are located at the promoters of highly expressed genes. **(7)** Instances corresponding to AP2-G motifs obtained in this study that are located at the promoters of highly expressed genes. **Table K.** Expression of multigene families and hypervariable gene families. List of expressed genes belonging to known multigene families previously described, potentially multigene families, and HVGs obtained by single-cell data. Columns include expression, ranking by expression (high, medium or low), functional annotation, orthology information, and production method.
(XLSX)
